# Therapy of *Helicobacter pylori*: Present Medley and Future Prospective

**DOI:** 10.1155/2014/124607

**Published:** 2014-04-01

**Authors:** Amin Talebi Bezmin Abadi

**Affiliations:** ^1^Department of Medical Microbiology, University Medical Center Utrecht, Heidelberglaan 100, 3584 CX Utrecht, The Netherlands; ^2^Department of Medical Bacteriology, School of Medical Sciences, Tarbiat Modares University, Tehran, Iran

## Abstract

The increasing prevalence of antimicrobial resistance has warned clinicians to adopt new strategies for dealing with the *H. pylori* infection. The success of various therapeutic regimens has recently declined to unacceptable levels. To date, first line therapies (including concomitant therapy and hybrid therapy), second line therapies (including bismuth-containing quadruple therapy and levofloxacin-containing therapy), and third line therapy (culture-guided therapy) had been introduced. In the near future, treatment of *H. pylori* is entering into a completely new resistance era. In this setting, despite the recent progress, we may only be targeting the patients with problematic *H. pylori*. Local preference for antibiotic selection should be an inevitable article in each therapeutic regimen worldwide. Meanwhile, improving the patients' compliance protocols and observed side effects in suggested therapeutic regimens should be considered cautiously. The new strategies in treatment should be adopted based upon local resistance patterns, which requires physician's resistance about the recommended guidelines. Designing new therapeutic regimen, which contains most effective available antibiotics with less possible side effects and high patient compliance, represents a challenging task in treatment of *H. pylori* infections.

## 1. Historical Therapy of* H. pylori*

*Helicobacter pylori* (*H. pylori*) is one of the most prevalent pathogens, which colonizes 50% of the world's population [1]. It is a spiral and microaerophilic bacterium that inhabits the mucosal layer of the gastric epithelium. The main* H. pylori* infection consequences can be listed as follows: gastroduodenal ulcer disease, chronic gastritis, and gastric adenocarcinoma. It has traditionally been confirmed that successful eradication of* H. pylori* can reduce the recurrence rate of duodenal ulcer, early gastric cancer, and metachronous carcinoma [2–4]. In fact,* H. pylori* is a persistent microorganism, which if not treated survives on the human gastric epithelial cells. Meanwhile, various research groups have failed to produce an effective vaccine that is quite preventive against* H. pylori* infection in various ethnic populations. With this regard, treatment seems as the first and obvious weapon to tackle this persistent infection. Usually, in the case of other infectious agents, eradication therapy results in nearly 100% successful rate, but the story is slightly different with* H. pylori* [5]. Many therapeutic regimens have been suggested, with different doses, durations, formulations, and exceptional drug administrations. Unfortunately, a universal therapeutic regimen to cure all* H. pylori* infections is not available [6]. Increased resistance rate, especially to the two major members of therapy (clarithromycin and metronidazole), has reduced the efficacy rate of those therapeutic regimens [7]. Optimistically, current cure rates with available therapeutic regimens are less than 80%, which call for urgent reconsideration about ongoing strategies on* H. pylori* and its therapy [7, 8]. Standard therapy (PPI, amoxicillin, and clarithromycin) is the most recommended/useful therapy to cure* H. pylori* infection; however, only a few recent studies showed acceptable efficacy rate [5, 6, 9]. As expected for a normal bacterial infection, monotherapy has been launched to eradicate the* H. pylori* from human stomach, but all hopes were almost fainted [10, 11]. Until now, some antibiotics showed a promising efficacy level for eradicating the infection by* H. pylori*; however, it seems unwise to rely on a single therapeutic approach [8, 11, 12]. In the past, clinicians checked susceptibility results before prescription; nowadays, they do not pursue this approach anymore. Meanwhile, diagnostic tests have been greatly improved, and clinicians now tend to use noninvasive methods for dealing with the gastroduodenal complaints [13–15]. Henceforth, lack of susceptibility tests resulted in scarcity of available data regarding antimicrobial susceptibilities. Indeed, underestimated value of providing* H. pylori* antibiotic susceptibility patterns is the Achilles heel of the new written guidelines [16–18]. Interestingly, using bismuth as a therapeutic drug against gastroduodenal complaints was a routine approach, although its mechanism of action is unclear [19, 20]. Using bismuth in European countries has been prohibited since a couple of years ago; however, keeping combined bismuth in designed therapeutic regimens of* H. pylori*, based on the latest published guidelines, remains controversial [17, 18, 21]. Among the bacterial infections in gastroduodenal route, only* H. pylori* can adopt such effective strategies to repel antibiotics from gastric epithelial cells [22]. Hence, one of the main causes of treatment failure is the evolved status of the* H. pylori* after several years living in the human stomach [8], which has led to the resistance phenotype against all three lines of therapies (first, second, and third) [23]. After a continuous series of therapeutic failures, several therapeutic regimens continue to be designed [24–26]. In the past, it was declared that* H. pylori* infection can be completely eradicated within a short treatment period [27]. Altogether, we are now entering a new era of the resistance which calls for new and smart strategies to deal with these* H. pylori* resistant strains [8]. Indeed, while a patient is factually cured with monotherapy, the likelihood of developing resistance against the other antibiotics increases, and this may result in multidrug resistant isolates [8, 17, 23]. However, it can be mentioned that skipping the antibiotic susceptibility testing, as proposed in the DANCE strategy, will facilitate emergence of antibiotic resistance [28]. It is likely that such general ideas on how to treat* H. pylori* can only reduce current efficacy of antibiotic therapy. As there are only a few available and effective antibiotics against* H. pylori,* and since combination of two or three antibiotics and an acid inhibitor is required, we should preserve our remaining treatment options. Yet, there are reliable methods to accurately determine presence of* H. pylori* in feces, saliva, and blood, such as ELISA and direct PCR [13]. As the molecular mechanisms of resistance of* H. pylori* are well characterized, determination of these genes with molecular tests based on* H. pylori* DNA in feces seems feasible [13]. At this time, a comprehensive strategy for dealing with the treatment of* H. pylori* infection is lacking. Furthermore, to date, status of retreatment guidelines for infected people with failed therapy is still under query. This paper discusses current available treatment strategies, as well as further steps and future prospects in treatment regimens against* H. pylori*. Additionally, we draw a real prospective for* H. pylori* therapy in the future.

## 2. First Line Therapy of* H. pylori*

**Figure 1:**
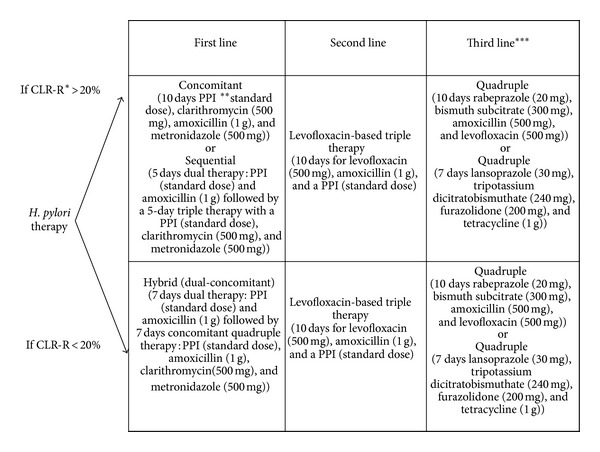
Recommended different therapeutic lines against* H. pylori *infection. *CLR R = clarithromycin resistance. **PPI = proton pump inhibitor. ***Standard empirical third line therapy is lacking.

Standard triple therapy (PPI, clarithromycin plus amoxicillin, or metronidazole) was the most useful regimen for* H. pylori* treatment in the past [18]. In case of failed first line treatment, second line treatment is chosen without resistance testing, whereas with further failure, third line treatment should be chosen only based on* H. pylori* cultures and antibiotic susceptibility testing (Figure 1).

Recently, the efficacy of triple therapy decreased globally due to the increased rate of clarithromycin resistance. In the early years of antibiotic resistance, standard triple therapy entitled as a best option for eradicating the* H. pylori* [29]. However, clarithromycin susceptibility disclosed a determining role for achieving the eradication of infection [9, 13, 30–32]. In other words, clarithromycin resistance is thought to be the main cause of eradication failure for standard triple therapy [8, 18, 33]. Nevertheless, current European guidelines suggest the use of 7 days of triple therapy in the regions where the rate of clarithromycin resistance is less than 15%; meanwhile, 14 days were recommended for areas with clarithromycin resistance 20% and more [17, 18, 21]. A randomized trial showed that aforementioned therapeutic regimen for 14 days resulted in an eradication rate of only 70% among the nonulcer dyspepsia patients, even as the rate for peptic ulcer patients is almost 82% [34]. Concomitant therapy is a novel regimen which is successful in the presence of clarithromycin resistance (Figure 1). This is a 4-drug regimen containing a PPI (standard dose), clarithromycin (500 mg), amoxicillin (1 g), and metronidazole (500 mg). This therapy is superior to standard triple therapy for* H. pylori* eradication [35]. Concomitant therapy is even less complex than sequential therapy as it does not require changing drugs during the treatment, whereas the better patient compliance can result in an effective therapeutic regimen. Recently defined, sequential therapy, concomitant therapy, and hybrid therapy are the novel introduced first line therapies [36]. Indeed, hybrid (dual-concomitant) therapy is consisting of a dual therapy with a PPI (standard dose) and amoxicillin (1 g) for 7 days followed by a concomitant quadruple therapy with a PPI (standard dose), amoxicillin (1 g), clarithromycin (500 mg), and metronidazole (500 mg) for 7 days [37]. However, extended duration of therapy up to 14 days in hybrid therapy and using three antibiotics in the last 7 days of the treatment are the main advantages than sequential therapy [37]. The current findings with sequential therapy are still inadequate to recommend it as first line therapy of* H. pylori* [36]. Prohibition of bismuth salts in some Western countries limits the use of quadruple therapy in those countries for first line treatment. Discovery of new antibiotics for use as first line treatment may still be an option. Clarithromycin-based design remains a major criticism of the standard triple therapy. In reality, increased rate of clarithromycin resistance in different regions of the world made the triple therapy less effective, at least for first line therapy [38, 39]. Actually, 13 years ago, a novel 10-day regimen was proposed, which was later known as a sequential therapy; it was a dual therapy (amoxicillin and PPI) given for the first 5 days, followed by a triple therapy (PPI, Clarithromycin, and Tinidazole) for the last 5 days [40]. Although meta-analysis proved that it can be even more effective than classic standard therapeutic regimen, data is only available from Italy [40]. Another concern about the first line therapy is the preference of the sequential regimens than standard treatment [41, 42]. Taking a lesson from other studies indicates that low efficacy of sequential therapy in patients with dual resistance (clarithromycin and metronidazole) can be a cautious consideration [43, 44]. Interestingly, Hsu et al. [37] reported a new sequential concomitant hybrid therapy with 99% eradication rate. However, this finding needs to be analyzed further in different geographical regions.

## 3. Second Line Therapy of* H. pylori*

After the first failed attempt at curing* H. pylori* infection, second line therapy was started. Basically, this therapeutic regimen is quadruple and it is used frequently as second line therapy [45]. Quadruple therapy is divided into two different regimens: with and without bismuth salt. Minakari et al. [46] reported another bismuth-based treatment including azithromycin, omeprazole, ofloxacin, and bismuth; however they failed to show the acceptable efficacy rate. Furthermore, in a study from Spain, 300 patients were analyzed for 10-day trial which showed 81% efficacy when they used levofloxacin-based therapy as second line treatment against the* H. pylori *infection [47]. The main skeptical point regarding the second line therapy is the increased rate of fluoroquinolone resistance among* H. pylori* strains globally during the years [48–50]. Noteworthy, safety of fluoroquinolones and levofloxacin is a limiting item in their application as the primary therapeutic line for* H. pylori* [51]. Undoubtedly, applications of initially prescribed antibiotics are not recommended in second line therapy. Furthermore, dose and duration of mentioned antibiotics are recommended to be checked cautiously in second line therapy [30, 36]. Due to the numerous indications on failures in first line therapy, we should be well prepared to design and think about new drugs as second line therapy. Several “rescue” therapies such as second line have been recommended yet, but still more than 22% of uncured patients emphasizes on therapeutic dilemma [52]. To date, some studies suggested the probiotics as additive to increase efficacy rate of recommended regimens in second line therapy [53], but continuous prescription of probiotics in therapeutic regimens of* H. pylori* infection requires more basic and clinical investigations. Collectively, a levofloxacin-based triple therapy might be an option to be prescribed as second line therapy worldwide.

## 4. Third Line Therapy of* H. pylori*

No validated empirical rescue regimen for the third line treatment of* H. pylori* infection is now available [54, 55]. More recent, both Maastricht III and IV consensus—2007 and 2012—suggest performing susceptibility tests before designing an effective regimen after the failures in first or second line of therapy [18, 21]. Currently, the main question mark regarding the third line therapy is referring to the various combinations of drugs and its optimal doses [54]. Actually, different alternative suggestions reported skyrocketing resistance rates against the antibiotics used, which became the main reason for treatment failure [56]. Since no well-known formulation has been established for third line therapy of* H. pylori* infection, different antimicrobial agents were examined, accordingly [57, 58]. The problem is that providing the preliminary data for susceptibility tests for the patients with failed therapy is not practically feasible. However, culture-guided therapy, which was examined by Cammarota et al. among 94 consecutive populations, can be a light in the darkness of treatment [59]. They reported >90% treatment efficacy when they prescribed regimen after performing the susceptibility test. More patients, particularly in various geographical regions, need to be examined for having a better conclusion out of this new approach.

### 4.1. Current Dilemma

The present lesson learned from existing challenges of* H. pylori* would be the global importance of the infection. However, the latest European consensus guideline is indicating on continuing usage of the quadruple therapy as first line of* H. pylori* therapy [17], an idea which disclosed limited options in* H. pylori* treatment. Actually, 20 years ago, when the first hints of* H. pylori* resistance emerged, clinicians underestimated the potential problem with* H. pylori* therapy. It appears that the difficulty of* H. pylori* therapy is relatively underestimated and thus needs to be considered as a special topic in current medical research [8]. Currently, in the case of* H. pylori* treatment, we have a different situation than 15 years ago. Overall, it is clear that the* H. pylori *should be considered as a superior infectious agent among human pathogens. Consequently,* H. pylori* infection should be addressed and cured as other bacterial infections accordingly [60].* H. pylori* eradication failure is an important problem that resulted in antibiotic resistance. Indeed, we are dealing with a sharp fall in effectiveness of different suggested therapeutic regimens. Notwithstanding, we only have a limited number of the antibiotics that can serve as different therapeutic regimens around the world. Meanwhile, a confused situation is observed with quadruple therapy [61, 62]. To date, the vast majority of studies did not examine the items such as: (i) combination of the drugs, (ii) treatment duration, and (iii) optimal dose among the different population. To be idealistic, current findings gently indicate that we are dealing with a “*superbug*” microorganism. First of all, the reconsideration for current adopted strategies for curing the* H. pylori *looks necessary. Clinicians tend to decline susceptibility tests before prescription of the antibiotics, a bitter fact that can harden the complexity of the current situation. Shortly after Maastricht meeting III at 2007, new calls for new consensus started [16]. It showed that even the new guidelines cannot meet all of the complexities. Indeed, using various antibiotics within the quadruple regimen will exacerbate the situation for patients who mostly suffer from other diseases. Regarding the number of the tablets administered over a short time, the quadruple therapy is prone to low compliance by patients, a problem which brings more thoughts to the mind before assigning the quadruple as a first line therapy. Epidemiological evidences indicate the significant association between gastroduodenal disorders and* H. pylori* infection [63–65]. Apart from various reports in different countries, it is safe to assume that we need to eradicate the infection in high risk population. At the moment, the query would be the determination of the high risk group among the different population worldwide. The first helping point can be determining current prevalence of severe gastroduodenal diseases in each country. Indeed, updated information about the susceptibility tests and prevalence of certain digestive diseases can help to design a treating panel of* H. pylori*. This suggestion needs a 10-year prospective study that can lead to the enough evidences to elucidate the (i) role of the* H. pylori* in occurrence of gastric cancer in scientific view and (ii) beneficial effects of* H. pylori* eradication to prevent gastric cancer in highly prevalent regions of the world. In other words, for regions with high prevalence of* H. pylori* and gastric cancer, we should design a same screening approach to find high risk population; thereby, we can draw a hypothesis for treatment of* H. pylori* infection. Until then, most of the suggested strategies cannot be easily generalized to other areas than primarily established.

### 4.2. Future Prospects

Undeniably, the* H. pylori* treatment story is far from complete. The current sharp fall in eradication rate of therapy has sobered to be careful in this topic. While the efficacy of first, second, and third lines of therapeutic regimens against the* H. pylori* is decreasing, a practical approach looks necessary for an urgent intervention. The historic therapeutic expectation for infectious agents is culture-susceptibility test in each patient. Undoubtedly, prescription based on local pattern of resistance would be an alternative for clinicians. In the past, we expected that new molecules would emerge as promising alternatives if routine therapy failed; unfortunately, we currently do not have that many proposed drugs as novel antibiotics for clinical practice [66]. Indeed, several studies showed that breakthroughs may occur if all considerations are taken into account properly [67, 68]. The high prevalence of* H. pylori* infection likely contributes to the fact that resistance can arise easily, a phenomenon which calls for continuous susceptibility tests (at least in pilot scale). However, development of molecular detection based on noninvasive methods is new and provides a definite and promising light for* H. pylori* therapy research. Moreover, due to the lack of hope for having several new antimicrobial agents in near future, we have to take care of the situation smartly. Vaccination was proposed for developing countries, though lack of progress diminished these hopes [69, 70]. Apart from different therapeutic regimens suggested, only few effective antibiotics remain an alarming status that calls for more thoughtful application of current available antibiotics. Hence, all decisions regarding the treatment in future should consider this limitation. Undoubtedly, the treatment protocols for the implementation of* H. pylori* eradication must be defined according to the common antibiotic medication or local antibiotic resistance and eventually the patient compliance. We now know that a successful treatment of* H. pylori* is a designation of all future formulation, which may be effective, inexpensive, and with less possible side effects during the therapy. Moving from regimens that contain two antibiotics to the therapies with three antibiotics, selected according to local high efficacy, will be one of the main inevitable* H. pylori *therapeutic strategies. An obvious consideration is to prevent the development and distribution of antibiotic resistance to other existing microorganisms. In the near future, eradication of* H. pylori* will come to a completely new stage. In this setting, despite recent progress, we only might target patients with problematic* H. pylori*. In keeping the current perception of* H. pylori* therapy, a new round of treatment failures would be possible. All new strategies should consider the point that we only have a limited number of antibiotics to use against* H. pylori* infection. In other words, we can introduce them into new therapeutic regimens based on local susceptibility tests. Undeniably, we should avoid losing current useful antibiotics by prescribing arbitrary regimens, which rely on unaware sensitivity pattern of the antibiotics. Overall, avoiding the antibiotic susceptibility testing will facilitate emergence of antibiotic resistance worldwide. In 2012, latest updated Maastricht guideline insists on antimicrobial susceptibility tests after failure in second line treatment [17]. In fact, we have to go further to find a solution for having valid data about the susceptibility results without doing gastroendoscopy. In other words, development of molecular detection of resistance provides a viable alternative. Certainly, endoscopy and subsequent culture and susceptibility testing are not always possible, while they are actually reliable methods to accurately determine presence of* H. pylori* (and its possible resistance) in samples from saliva, blood, and feces via direct PCR. Conclusively, the aforementioned problems call for urgent attempts to suggest an empirical therapy based on local resistance patterns. Indeed, we should identify the* H. pylori* positive individuals who first suffer from problematic digestive disorders and secondly require a therapy. Due to high prevalence of* H. pylori* infection and those linked digestive diseases in different regions, all established guidelines require governmental approvals and positive signals from insurance companies before becoming acceptable adopted strategies in different countries. Hence, new strategy toward the* H. pylori* treatment should be taken under the assumption for reaching the 100% expected rate of eradication. According to this new proposed strategy, it appears that most of current available therapeutic regimens seem unprofitable due to the low efficacy rate. Designing a new therapeutic regimen which contains most effective available antibiotics with less possible side effects and high patient compliance would be the most challenging topic in* H. pylori* future prospective.
